# GP speciality training in areas of deprivation: factors influencing engagement. A qualitative study

**DOI:** 10.3399/bjgpopen19X101644

**Published:** 2019-05-15

**Authors:** Marianne McCallum, Sara MacDonald, John McKay

**Affiliations:** 1 GP Clinical Academic Fellow, General Practice and Primary Care, Institute of Health and Wellbeing, Glasgow University, Glasgow, UK; 2 Senior Lecturer in Primary Care and GP Clinical Academic Fellow, General Practice and Primary Care, Institute of Health and Wellbeing, Glasgow University, Glasgow, UK; 3 GP Assistant Director, Quality Improvement and Performance, Medical Directorate, NHS Education for Scotland, Glasgow, UK

**Keywords:** Postgraduate education, Inequalities, Family medicine, Primary care, General practice, Socioeconomic factors

## Abstract

**Background:**

GP training practices are less likely to be situated in areas of deprivation; little is known about GP views of postgraduate training in such areas.

**Aim:**

To explore the views of GPs working in deprived areas about GP speciality training (GPST).

**Design & setting:**

Qualitative in-depth interviews with GPs working in practices in deprived areas in Scotland.

**Method:**

Ten in-depth interviews were conducted with GPs in training and non-training practices, to explore views on training. Interviews were audiotaped and transcribed verbatim, and inductive thematic analysis was undertaken.

**Results:**

The importance of producing ‘well-rounded’ GPs who are able to work in a variety of environments was highlighted. Trainees need exposure to the specific challenges of deprived contexts (such as early multimorbidity, child protection, and addiction) and the benefit of this for trainees was thought to be invaluable. GPs identified many perceived barriers and benefits to training, some generic but some — such as inspiring the next generation (benefit) or overwhelming workload (barrier) — may be more relevant in areas of high deprivation. Overwhelming workload was the main reason for not becoming a training practice, though some would consider it if supported to develop a training culture. All the GPs, including non-trainers, were involved in optional activities which were felt to be important for resilience.

**Conclusion:**

GPs in areas of deprivation highlighted specific skills that could be gained by undertaking at least a part placement in deprived areas, with different skills likely to be gained from affluent areas. National education bodies should consider GP training rotations ensure a variety of training environments.

## How this fits in

GP practices in deprived areas are proportionately less likely to become training practices, and there is a paucity of literature looking at the views of GPs who work in these areas on training.

Most of the benefits and barriers to training in deprived areas are similar to those already identified in the wider literature, and the main barrier to training is GPs already feeling overwhelmed by current workload.

The key benefit identified was for trainees and was exposure to the medical and social complexity experienced in these areas, which the GPs in this study felt all trainees should experience.

This study suggests that supporting keen practices to embed a training culture may help increase training practice numbers in these areas, and that GPs in these areas use optional activities, including training, to help prevent burnout.

## Introduction

In the UK, GPST usually involves a 3-year training programme with 18 months of this programme spent in an approved GPST practice supervised by a named GP educational supervisor. In Scotland, there are significantly more GPST training practices in affluent areas than deprived;^1,2^ in England, training practices are under-represented in ethnically diverse inner-city areas;^3,4^ and in France, training practices have fewer low-income patients than the national average. Size is a significant predictor of training status:^1^ in Scotland, practices in deprived areas tend to be smaller.^5^

A recent review of GPST recognised trainees needed to experience different types of general practice to gain additional, wider skills.^6^ A recent survey of GP trainees in Yorkshire and Humber suggested that most trainees were keen to work in areas of high socioeconomic deprivation.^7^

There are some post training posts, such as the Glasgow Deep End pioneer^8^ or the Yorkshire and Humber trailblazer,^9^ which offer support for recently qualified GPs, but these will not be available to the vast majority of newly qualified GPs, and do not address the potential lack of exposure during training. The relative lack of access and exposure may result in GPs who lack confidence or competency in dealing with the complex health and social issues that deprivation brings: higher levels of addiction and child protection; dealing with third sector agencies; and managing multimorbidity in the context of social complexity.

Trainees are more likely to practice in areas similar to those where they train;^10,11^ thus, lack of exposure to areas of social deprivation during GP training may affect future recruitment. While the influences on future career choice are many, experience in GP training has a role.^11^ Postgraduate programmes that focus training on underserved areas may increase the proportion choosing to work there,^12–14^ and there is some evidence that exposure to training in inner city areas may increase the likelihood of doctors choosing to work there.^10,12,13,15^ The inequity of training practices across the social gradient could, arguably, be viewed as a further manifestation of Tudor Hart’s Inverse Care Law.^16^

Multimorbidity increases and starts at an earlier age as socioeconomic deprivation increases.^17^ Despite increasing morbidity and mortality, there is no increase, in Scotland, in the number of GPs per head of population^18^ and, in England, there are fewer GPs working in more deprived practice areas compared to more affluent ones.^19^ In areas of social deprivation, people present with more problems in shorter consultations, resulting in increased GP stress,^20,21^ increased pressure, and ultimately, unmet need. In Scotland, the average age of GPs in deprived areas is higher,^22^ suggesting that proportionally more GPs will retire in the next decade. This will potentially worsen the levels of provision of GPs in areas where recruitment is challenging and needs are complex.^22^

These issues point to a pressing need to encourage GPs in deprived areas, and their associated practices, to undertake GPST. Although size of practice can influence a partnership’s decision to train,^1^ many small practices in very deprived areas undertake successful GP training. It is likely that size is not the only limiting factor for practices choosing to train. A search of the literature revealed some studies on benefits and barriers to training under and postgraduates,^23–31^ but only one report looking particularly at the situation in deprived areas.^32^

Given the importance of having a workforce exposed to the multiple different social environments that GPs practise in, the policy initiatives to attract GPs to deprived areas, and the desire of GP trainers to provide training in multiple environments,^22^ it is important to understand both the views of those who undertake GP training and those who do not regarding the benefits and challenges of undertaking training.

The aim of this study was to explore the views of GPs working in deprived areas (from training and non-training practices) on benefits and barriers to GPST, those thought to be specific to areas of deprivation, and how barriers could be overcome.

## Method

### Study design and setting

Qualitative in-depth interviews explored GPs’ (from training and non-training practices) views on training. The interviews were conducted with GPs from across Scotland working in practices serving areas of high socioeconomic deprivation.

### Sampling

The Scottish Index of Multiple Deprivation (SIMD) 2012^33^ identifies small areas (data zones) that measure concentrations of multiple deprivation across all of Scotland in a consistent way. It then ranks these from most deprived (ranked 1) to least deprived (ranked 6976). NHS Scotland Information Services Division (ISD)gp^5^ links the Community Health Index (a unique patient identifier) of every patient in each general practice in Scotland to their postcode. Each patient is then mapped to an SIMD data zone based on the postcode they reside in. The complete list of data zones is ranked and divided into quintiles, matching each patient to a deprivation quintile. The number of patients in each quintile in a practice is displayed annually by ISD and is publicly available.^5^ This routinely-collected information was used to identify a target population of GP practices with high blanket deprivation (that is, a high proportion of patients living in the most deprived quintile),^34^ or a high global deprivation score^2^ (an average score based on the proportion of patients in each of the five deprivation quintiles ranging from 1, most affluent, to 5, most deprived). A training database held by NHS Education for Scotland (NES) was used to clarify which of these practices were training practices. The role of NES is described in Box 1.

Box 1. **The role of NHS Education for Scotland (NES) in postgraduate GP training**

**Table:** 

NES is an NHS Scotland board with national responsibility for GP postgraduate training.Applicants apply to train in the Scottish deanery; if accepted, they can specify one of four regions within which they wish to train. Each region has several training programmes, each with their own training programme directors: West Region: the largest region, covering Greater Glasgow, South West Scotland, and much of the central belt, including Stirlingshire. South East Region: covers Edinburgh, West Lothian, and the South East of Scotland, including some of Fife. East Region: covers the cities of Perth and Dundee, as well as wider Tayside and some of Fife. North Region: covers the cities of Aberdeen and Inverness, and most of the highlands.

Box 2. **Summary of ‘well rounded GP’ theme**

**Table:** 

**Able to work in wide variety of clinical environments:** *‘I just think you are in danger of operating in a small number of gears if you don’t get some training in an area of deprivation as well, you just don’t have just such a big big range.’* (GP 7, training) *‘ I would go so far as to say that if you, if you train in a practice like this, you can work anywhere, if you’ve trained in a middle-class, better off, more affluent practice, you’d struggle in a practice like this. We’ve had some evidence of that from some of the locums we’ve employed where they’ve come from, they’ve trained in a comfortable, middle-class area and they come here and it’s really, wow …*’ (GP 3, training) **Exposure to specific challenges, during training, from deprived contexts:** *‘ … but I do think that people should have exposure to both, because I’ve worked in a posh area and I’ve worked here as well, and you do change your consulting style and say different things, so I think they need to be rounded to do a bit of both.’* (GP 9, non-training) **Importance of exposure, as many medical students come from middle-class backgrounds:** *‘Certainly, a lot of doctors come from a kind of stable background, they may come from slightly higher social class, em, they may not, you know, have had much exposure to the things that are going on.*’ (GP 3, training) **Experience in more affluent areas important too:** *‘And there are some skills in dealing with more, perhaps, articulate demanding patients, you know, we are at one end of the spectrum and to balance with the other end is probably good*.’ (GP7, training)

To provide a geographical spread, a sampling framework purposively sampled practices from all four regions of the NES Scottish deanery. As the majority of areas of deprivation in Scotland are in the West region, proportionately more practices from this region were interviewed. Of 15 practices from the sampling framework contacted by email (to either the doctor or the practice manager), 11 responded and 10 were able to go on to full interview in the study time period. In the training practices, the main trainer or educational supervisor was interviewed; in non-training practices, a GP partner.

### Data collection

Semi-structured interviews explored opinions on training, including the benefits and barriers, for trainees, trainers, and the wider practice team. The topic guide (available from the authors on request) was based on themes from a focus group discussing GPST in deprived areas, published in 2010.^32^ This was the only literature found specifically looking at training in deprived areas. An iterative approach adapted the schedule as the interviews were conducted. All interviews were carried out by one author between January–May 2017, lasted between 40–65 minutes, and were audiotaped, anonymised, and transcribed verbatim.

### Data analysis

All the transcripts were read and analysed, using line-by-line coding, by the interviewing author. Inductive thematic analysis was used to identify key themes and subthemes.^35^ Two other authors also independently read the transcripts to authenticate themes. Any disagreements in coding were discussed and clarified, and agreement was reached by all authors. The summarised coding tree is available from the authors on request. Themes regarding benefits and barriers already identified as relevant to all training practices (many based on teaching undergraduates/Foundation Year 2 [FY2] placements)^23,26,27,29,31^ in the literature^23–31^ were classified as ‘generic’. Benefits identified in the literature included keeping clinical knowledge up to date,^23,26,30^ learning from trainee,^30,31^ personal fulfilment,^25,30^ increased practice morale,^23,26,29^ enjoying teaching,^26,27,30,31^ the trainee–trainer relationship,^24^ GPs passing on their skills,^30^ future workforce planning,^25^ and promoting general practice as a career.^30^ Barriers included time,^24,26,27,30,31^ space,^26,30^ workload,^26,30^ and finance.^24,30^ Remaining benefits and barriers identified were classified as either ‘universal but more pertinent in deprived settings‘ (based on increased unmet need), or ‘particular relevance to deprived settings’. The classifications were discussed and agreed by two authors.

## Results

A summary of the characteristics of the practices selected are shown in Table 1 . Data saturation was reached at nine interviews with no new themes identified from the tenth interview. Three key themes emerged from the data regarding GP speciality training in deprived areas and are expanded on below.

**Table 1.** Table summarising characteristics of study practices with comparison, where relevant, to Scottish average

Characteristics	
**Total participants,** ***n***	10
Trainers, *n*	5
Non-trainers, *n*	5
**Training practices in NES Scotland deanery, *n***	5
**Location (region of deanery**)	
West, *n*	5
East, *n*	2
South East, *n*	2
North, *n*	1
**Average list size per practice, *n***	5620
Scotland average, *n*	5710
Range in practices interviewed, *n*	2827–9118
**Proportion of practice list in the most deprived quintile (2015),** %	63.61
Scotland average, %	16.2
Range in practices interviewed, %	25.6–87.9
**Average deprivation score^a^**	4.54
Scotland average	3.11
Range in practices interviewed	3.95–4.81

a Deprivation score weighted by the proportion of the practice in each of the five deprivation quintiles: 1 (most affluent) to 5 (most deprived). For example, Practice 8 has a lower proportion of patients in quintile 1, but has a higher score as almost all their patients live in postcodes in the two most deprived quintiles.

### ‘Well rounded’ GPs

All the GPs felt strongly that GP training should produce ‘well-rounded’ GPs (Box 2) with the skills to work in a wide variety of clinical environments; exposure to a range of environments during training was therefore critical. They felt that the context in which they worked provided specific challenges and experiences that all trainees should be exposed to. In particular, due to the high levels of deprivation they worked with, the practice and consultations had been adapted to manage the complexity they faced. They felt experiencing this provided trainees with useful skills, regardless of the type of practice they worked in long term. Several GPs also felt this was particularly important as doctors are more likely to come from middle-class backgrounds,^36^ and may not truly understand the reality of many patients’ lives and, therefore, the barriers they may face.

### Benefits and barriers to training

**Figure 1.**
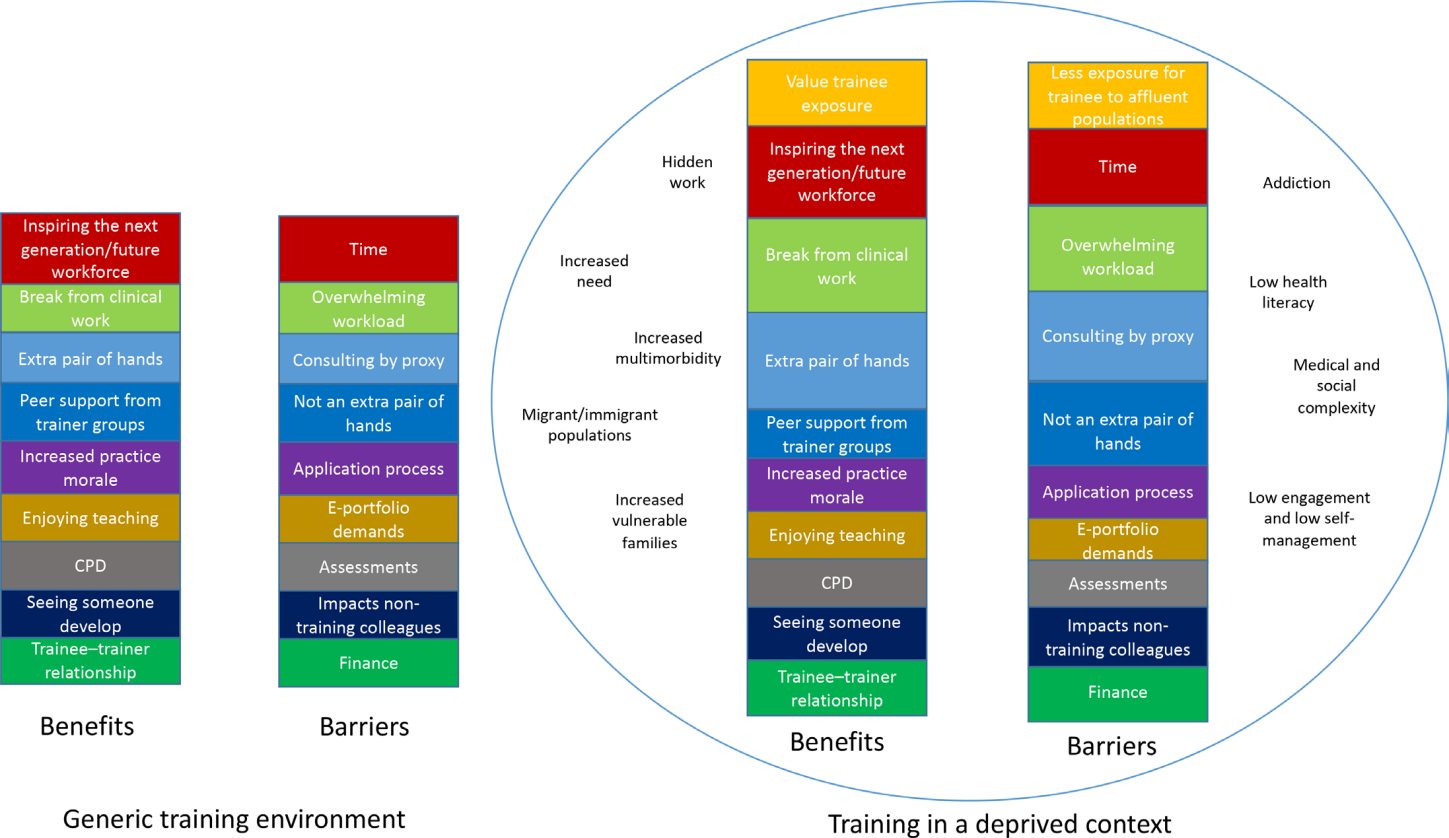
GPs’ perceptions of the benefits and barriers of training, and how these were affected if training occurred in the context of deprived areas

Figure 1 summarises the GPs’ perceptions regarding benefits and barriers to training, and their view of the impact of deprivation on these benefits and barriers.

#### Benefits

The GPs identified all the benefits previously mentioned in the literature and, in addition, the trainers identified peer support from other trainers as a key benefit for them. The key benefit of training in a context of high deprivation, identified by all the GPs, was the benefit to the trainee of exposure to the specific challenges more prevalent in these areas. Specific examples given were addiction, child protection, managing complex multimorbidity (particularly at an earlier age) in the context of social complexity, learning to manage patients with lower health literacy, using interpreters, and having to deal with other health and social care professionals. Although some practices felt the trainee provided an extra pair of hands, welcome in their busy context, others felt that the work required to supervise and train meant that, overall, it was a neutral effect. Box 3 summarises the benefits of training identified by participants.

Box 3. **Summary of themes regarding benefits of training, categorised as generic, universal but more pertinent in deprived areas, and of particular relevance to deprived areas**

**Table:** 

**Benefits generic to training**
**Trainee–trainer relationship:** *‘It can be rewarding and stimulating. It’s persistent learning from each other, so we learn from the trainees, they learn from us.*’ (GP 1, training) **CPD and learning from the trainee:** *‘I’m picking up stuff from them all the time, new developments, new treatments, stuff like that, stuff they’ve read, so I’m … I get a lot personally, em, professionally, I get a lot of, I think my professional performance is improved by exposure to junior doctors.’* (GP 3, training) **Passing on skills and enjoying teaching:** *‘I hope that I have experience and skills that are useful to pass on to others and that could help to nurture them … It does have its moments, if you get someone who’s got challenging problems, but on the whole I get a buzz out of it.*’ (GP 4, training) **Increased practice morale:** *‘ … the most important bit is the ethos of the practice being open, that we are happy to review our own clinical practice as partners but also the admin team, that they know how to support these young trainees*.’ (GP 1, training) **Value of peer support from trainer groups:** *‘I think it is important to meet other colleagues if you can, and as much as I am really fond of all of my partners, it’s good to meet colleagues in other practices. You know, just for interest and comparison and, yeah, just as another reference point.’* (GP 7, training)
**Universal benefits, but more pertinent in deprived areas**
**Trainees as an extra pair of hands:** *‘ … for your more challenging patients they may see the registrar for a period of time and you can get that bit of a break. And there is an awful lot to be said for that, to be keeping going*.’ (GP 8, training) **Future workforce planning:** *‘I would want to encourage people to put their energies and efforts into an area of deprivation, if possible.*’ (GP 7, training) **Inspiring the next generation:** *‘It’s part of why I want to do it as well, I suppose, you know, I don’t want to see general practice fizzle out and die because I think it’s brilliant and really, really important.*’ (GP 6, non-training but hoping to train)
**Benefits of training in deprived areas**
**Value of exposure:** **Exposure and management of challenges more common in deprived areas:** *‘So, I do think there is something about being exposed to the problems, and having to solve those problems, or at least ameliorate those problems, which is valuable to, eh, a young registrar.’* (GP 3, training) **Multimorbidity (earlier age and later presentations):** *‘ … when people present, the degree of illness they have is often hugely different from what you would do in an affluent area. They are usually more diseased, more if that … if you understand what I mean. So that when they present, they present further down the line.’* (GP 9, non-training) **Managing illness in the context of social complexity:** *‘They also learn, um, complexity … and they also learn the whole benefits system, social issues like child protection and lots of palliative care as well, because we have so many people basically dying. And especially sudden deaths, so our rate of sudden deaths is higher than the average, so they learn how to deal with the procurator fiscal, so overall I think they get a very wide spread of clinical exposure … they might be missing the worried well*.’ (GP 1, training) **Low health literacy:** *‘Yes, I mean, a huge number of our patients can’t read or write, so the spoken word, you still have to use very simple language with them, so it’s a different way of communicating. You can’t just hand a leaflet out*.’ (GP 10, non-training)

#### Barriers

The barriers of time, workload, and finance identified in the literature were all mentioned by the responders, as were some specific barriers related to space, the training process, or the application (these last three were felt to be surmountable). The main reason given for not training was overwhelming workload, which responders felt was an issue for all GPs, but was particularly pertinent in their areas.

Poorly performing trainees are a barrier for all practices, and can be a stressful experience for all involved. However, the GPs spoken to felt this was of particular importance in their areas because of the added complexity (social and medical), and therefore the added worry for trainers that things were being missed. Responders also felt the increased complexity of their patients meant that all trainees required extra supervision, which some of the responders felt led (or would lead) to extra stress. One non-training GP spoke of bad experiences with FY2s whom she and her partners felt did not see, or appreciate, the extra work needed to support compliance, think about transport, or consider where and why extra safety netting was required. This resulted in increased work for the whole practice and increased anxiety for the partners, and was a key reason why they would not want to train. As the GPs felt that poorly performing trainees were more of an issue in their context, this theme was classified as more pertinent in deprived areas. Box 4 summarises the perceived barriers to training.

Box 4. **Summary of themes related to barriers of training categorised as generic, universal but of more value in deprived areas, and specific to deprived areas**

**Table:** 

**Generic barriers to training**
**Space:** *‘You need the space as well, and it used to be in our old surgery we were limited in space … we have a bit more space when* [area X] *health centre was done years ago so we got a bit more space. But I’m sure we would have worked round the space side of things, so it was more the physical capacity of GPs*.’ (GP 2, non-training) **Application process:** *‘Em, the paperwork I think, I’m sure you’ve have heard it said is quite onerous. In some ways I think it needs to be, em, particularly for that first assessment. I suppose I would slightly question whether they need as much paperwork and proof once you have been approved at the beginning but, obviously, things can slip so I understand the need for quality*.’ (GP 7, training) **E-portfolio and assessment demands:** *‘ … then, obviously, the assessments dominate our tutorial time, um, so I feel we are having less time to teach, especially if you are the lead trainer who does most of the assessments*.’ (GP 1, training) **Impact on non-training colleagues:** *‘I suppose the only thing I would say is, at times, there has been some tensions in terms of partners questioning the time needed for training. It has been a source of tension at times, and discussion.’* GP 7, training) **Finance:** *‘ … suppose money can be an issue, not that it ever was with us, but I think sometimes people are undervalued with the time that they spend and I’m not really sure, and I go to all these meetings and there is backfill for locums, but it only really pays your locum*.’ (GP 10, non-training)
**Barriers more pertinent in deprived areas**
**Time:** *‘ … just patient’s ability to comply with systems and medication compliance, so I think though it’s very difficult to demonstrate, just the day to day work, we are busier than those in practices where the patients are much more self-reliant, much more self-starting, much more able to navigate the systems, know where to go and when to go. So, that doesn’t leave us as much extra time, or as much slack in the day to undertake training. Em, and certainly in this practice, it’s the time issue that stops us considering being a training practice*.’ (GP 5, non-training) **Overwhelming workload:** *‘ … think a lot of practices in deprived areas just feel they’re creaking at the seams and they can’t see enough patients because patient demand, you know. Research has shown the number of times your average patient sees the doctor is much higher in a deprived area than it is in a more better off area*.’ (GP 6, non-training) **Trainees not an extra pair of hands or requiring extra supervision:** *‘Because it’s not just the medical problem, it’s the whole patient context. Which I think we found the junior staff were just not grasping, and all the additional things like communication with chemists, compliance devices for medication, em, liaison with different agencies, they just didn’t know about. So you were almost doing it all over again on their behalf, so actually we didn’t find we were given any extra time at all.*’ (GP 5, non-training) *‘So we have to protect them, especially at the beginning of their training, um, trying to look a bit what kind of patients are being booked in. Admin team needs to be on top of that, if they see that, um, for example, for emergencies that they protect the trainee a bit. And supervision, I think, has to be much much closer because, um, the risk is so much higher in an area like ours.*’ (GP 1, training) **Poorly performing trainees:** *‘ … um, we absolutely need, um, fairly good and solid GP trainees. We could not cope with an underperforming trainee, um, because of the complexity of the patients, um, and it can be very challenging even with good trainees. Because there is such a complexity to the patients that you can easily feel overwhelmed.’* (GP 1, training)
**Barriers specific to deprived areas**
**Less exposure to better educated patients:** *‘ … think the worried well, we have some, so it’s not all deprived, but not nearly as much and actually, the potential straightforward who have condition A: for condition A to get better you need to do plan B and please go away and do plan B … oh okay you are going to do plan B. And I think that they probably wouldn’t get that, and I think they would miss out on the very informed patient who knows exactly, or who knows exactly what they want with their illness, who researched it all, and and … sharing decision-making.*’ (GP 8, training) **Busyness discouraging trainee (mentioned by two responders**): *‘I would be concerned about a trainee only training in this area of becoming discouraged actually, em, ‘cause even for we who are used to it, it often feels like wading through treacle, like you are getting absolutely nowhere. And I would be concerned for a young doctor that that might be a bit overwhelming.’* (GP 5, non-training)

### Overcoming barriers to training

The current overwhelming workload was the main reason practices gave for not training, and it was also recognised as an issue for practices currently training. However, despite workload and pressure being listed as a reason not to train, all the non-training GPs interviewed were also involved in optional additional professional activities. These included being an appraiser, teaching medical students (at university or within their practice), or involvement with the Local Medical Committee. All the GPs spoke of how clinical contact generated work, and that having some time away from this was seen as essential to prevent burnout:

*‘Yes, I think, for me, it’s actually very nice to do something which, compared to practice work, is almost stress free* [laughs] *You know, it’s great. And the task is contained when you’ve finished teaching, the kind of teaching I do, you turn off the computer and the lights and it’s finished, done, you drive away and that session is done. There is nothing following it, no one is going to phone, you know there’s no letters, no one is going to become ill overnight. So it’s great, it’s brilliant, it’s like liberation. Likewise, with appraising, it’s a contained task, once you’ve done it you’ve done it and that’s it, and I find that really helpful that at least part of my work is like that.*’ (GP 5, non-training)

The non-training GPs used their other non-clinical commitments, which they felt were not as time-intensive as training, to provide a natural break. The value of optional activities in potentially building GP resilience is not something previously identified, but these findings suggest GPs are using optional activities to balance their clinical workload, and this could be promoted as a potential benefit in GPST.

Interestingly, most of the training practices spoken to had a longstanding ‘culture of training‘ within their practice from before the increase in workload over the last 10 years. The one practice that had become a training practice in the last 10 years had significant support from their local health board, and NES to do so:

*‘The fact that I got support from my clinical director made a big difference. My clinical director was a trainer and his line was “if we get people down here and train them here, they’re more likely to stay here*.”’ (GP3, training)

All but one of those who were not training would be interested in it. They recognised that once they had set up a training culture, maintaining it might be more manageable, but they could not see a way to get there with their current workload:

*‘Felt we would have needed, look you know what would have helped, you know if there was some sort of locum provision or anything like that. But we’d have had to bring in locums or something to cope with it initially*.’ (GP2, non-training)

This suggests that support to create a culture of training within keen practices may be one way to increase training numbers in underserved areas. However, the GPs who were interviewed had all responded to the request to take part, therefore this group may be self-selecting and not representative of non-training practices in general.

## Discussion

### Summary

GPs in deprived areas felt training should produce ‘well rounded GPs’, who were confident and capable of dealing with many of the specific issues faced in areas of high deprivation (in particular addiction, child protection, dealing with third sector agencies, and managing multimorbidity in the context of social complexity). They also felt that experience during training of how practices adapt to work with high levels of deprivation could provide useful skills for dealing with any patient, regardless of the type of practice the trainees work in long-term.

Many of the benefits identified from undertaking speciality training were similar for all practices involved in training, regardless of social context, although inspiring the next generation and future workforce planning were identified as particularly pertinent in deprived areas. The main benefit of training in areas of high deprivation was trainee exposure to the medical and social complexity that patients in these areas experienced.

The key barriers to training were increased workload and lack of time. Both trainers and non-trainers alike also identified that, due to increased medical and social complexity, increased support and supervision was needed, requiring more work for trainers.

This is a small sample, but most of the non-training responders would like to consider training and are already voluntarily involved in optional activities, seen by participants as essential in allowing them to manage their heavy clinical workloads. From this small group, it appears targeted support to help embed a training culture by freeing up GP time, and potentially promoting the benefits for reflection that training brings, may help some practices to start training.

### Strengths and limitations

This is the first study the authors are aware of that has sought the views of GPs from training and non-training practices working in areas of high socioeconomic deprivation. A key strength is that GPs were interviewed from across all four geographical regions in Scotland, and the universal experience and challenges of practising and training in areas of high deprivation were consistently identified. The common challenges to practising in such environments therefore appear to be the consequence of deprivation and apply regardless of geography. Although pertinent to the Scottish context, the consistency of these findings suggests they are likely to be relevant to similar populations.

A key limitations of this study is its size. Although data saturation was reached, that may be because of the self-selecting nature of the sample of GPs who volunteered to be interviewed. The findings suggests that there are some practices that are keen to train, and who may be able to do so with support, but the numbers are too small to make a definite general statement. Further work surveying a wider range of GPs would be required to confirm these finding.

A further limitation is that, due to time constraints, only trainers in deprived areas were interviewed; it would be helpful to do the same exercise in more mixed and affluent practices to see whether findings differed.

### Comparison with existing literature

The perceived importance of GP trainees experiencing a variety of populations during training expressed by participants in this study echoes current recommendations,^6^ as well as the views of GPs who work in both affluent and deprived areas.^22^ This is particularly important given that medical students are predominantly from more affluent backgrounds.^36^

Literature shows GPs are motivated to teach because it keeps their clinical knowledge up to date,^23,26,30^ they learn from their trainee,^30,31^ they receive personal fulfilment,^25,30^ it increases practice morale,^23,26,29^ and they enjoy teaching.^26,27,30,31^ The trainee–trainer relationship is highly valued,^24^ and GPs value passing on their skills^30^ and promoting general practice as a career.^30^ They also feel a responsibility to their profession to train,^24,25,30^ and feel it helps with future workforce planning.^25^ All of these, except a responsibility to train, were identified by the participants in the interviews, confirming that many of the benefits of training are universal. The participants also identified support from other trainers as a benefit. Barriers to GP training reported in the existing literature include time,^24,26,27,30,31^ space,^26,30^ workload,^26,30^ and finance,^24,30^ all barriers identified by the participants. Recent literature has shown that the current pay-for-performance model in England means GPs in deprived areas are paid less despite having a higher workload.^37^ Despite this, and although it was mentioned, finance did not appear to be a significant issue for the GPs interviewed. The trainers who did mention it felt that, as the trainer’s grant had not substantially increased, training was cost-neutral for the practice. The non-trainers felt the main reason they were not training was due to workload, and additional finances would not change that.

One key theme, not mentioned in the literature but important to the interviewees, was the perceived need for increased supervision and support in their context because of high levels of medical and social complexity. This suggests that in deprived areas, the increased supervision required for more complex patients means the benefit to the practice of a trainee may be perceived as more limited.

Multimorbidity increases in prevalence and starts at an earlier stage as socioeconomic deprivation increases.^17^ Despite this, there is a flat distribution of GPs, and the potential increased need and workload is not matched by any extra GP resource.^26^ People present with more problems in shorter consultations, resulting in increased GP stress.^18,20,21^ This study suggests this increased unmet need particularly impacts practices in deprived areas in terms of considering training, and it may also require them to have increased support and supervision in place when trainees attend their practices.

Previous work has also suggested that a significant proportion of non-trainers would want to train, but that the main barrier is time.^28^ These results are borne out by the people questioned in this study. Although all the non-trainers were involved in optional professional activities, these were often not seen to be as time-intensive as training. Some GPs had experience of undergraduate teaching, which was universally enjoyed, and FY2 teaching, the experience of which had been mixed. This may be a step for practices interested in training in the future, but the numbers in the study are too small to be definitive.

### Implications for research and practice

There are specific GP skills (examples mentioned by participants included increased exposure to managing addiction and child protection; understanding the benefits system; managing migrant populations; and how the social determinants of health can impact patients directly) to be gained undertaking at least some time in a placement in a deprived area, and national education bodies should consider this in GP training rotations. Practices may need to provide increased supervision and support for trainees in these areas due to increased medical and social complexity; training bodies may wish to actively look at how they may support this.

Although deprivation was the focus of this study, further work that also interviewed trainers working in affluent areas would be of value to clarify their experience, and to see whether there are different supervision requirements that, in turn, may have resource implications.

This study suggests that there are non-training practices in deprived areas which are keen to train. These results could inform future national initiatives that encourage GPs who practise in areas of deprivation to undertake speciality training accreditation as an educational supervisor by stressing the positive outcomes of training across practice teams. Targeted interventions that simultaneously emphasise benefits and address barriers by providing discreet support would allow GPs and their colleagues in smaller practices in areas of deprivation^38^ to achieve training status. The new GP contract in Scotland, which aims to address workload issues and the role of GP as an expert generalist, provides an opportunity for change and may offer additional benefits for the future of GP training in areas of deprivation.
